# Genomic and transcriptomic analysis of pituitary adenomas reveals the impacts of copy number variations on gene expression and clinical prognosis among prolactin-secreting subtype

**DOI:** 10.18632/aging.202304

**Published:** 2020-12-19

**Authors:** Yiyuan Chen, Hua Gao, Weiyan Xie, Jing Guo, Qiuyue Fang, Peng Zhao, Chunhui Liu, Haibo Zhu, Zhuang Wang, Jichao Wang, Songbai Gui, Yazhuo Zhang, Chuzhong Li

**Affiliations:** 1 Department of Cell Biology, Beijing Neurosurgical Institute, Capital Medical University, Beijing 100070, China; 2 Department of Neurosurgery, Beijing Tiantan Hospital, Capital Medical University, Beijing 100070, China; 3 Department of Neurosurgery, The First Affiliated Hospital of Zhengzhou University, Zhengzhou 450052, China; 4 Department of Neurosurgery, People's Hospital of Xinjiang Uygur Autonomous Region, Xinjiang 830001, China; 5 China National Clinical Research Center for Neurological Diseases, Beijing 100070, China; 6 Brain Tumor Center, Beijing Institute for Brain Disorders, Beijing 100070, China

**Keywords:** pituitary adenoma, WGS, CNV, bromocriptine resistance

## Abstract

Pituitary adenomas (PAs) are slow growing and benign primary intracranial tumors that often cause occupying effects or endocrine symptoms. PAs can be classified into various subtypes according to hormone secretion. Although widespread transcriptional alterations that cause aberrant hormone secretion have been characterized, the impact of genomic variations on transcriptional alterations is unclear due to the rare occurrence of single-nucleotide variations in PA. In this study, we performed whole-genome sequencing (WGS) on 76 PA samples across three clinical subtypes (PRL-PAs; GH-PAs, and NFPAs); transcriptome sequencing (RNA-seq) of 54 samples across these subtypes was also conducted. Nine normal pituitary tissues were used as controls. Common and subtype-specific transcriptional alterations in PAs were identified. Strikingly, widespread genomic copy number amplifications were discovered for PRL-PAs, which are causally involved in transcriptomic changes in this subtype. Moreover, we found that the high copy number variations (CNVs) in PRL-PA cause increased prolactin production, drug resistance and proliferative capacity, potentially through key genes with copy number amplification and transcriptional activation, such as BCAT1. This study provides insight into how genomic CNVs affect the transcriptome and clinical outcomes of PRL-PA and sheds light on the development of potential therapeutics for aberrantly activated targets.

## INTRODUCTION

Pituitary adenomas (PAs) are primary intracranial tumors that occur in 0.1% of adults [1, 2]. PAs can be classified according to the presence of abnormal hormone secretion (functional PAs, 36%-54%; clinical nonfunctional PAs, NFPAs, 46%-64%) [3, 4]. Functional PAs can be further divided into subtypes according to hormone secretion status, and prolactin-secreting PAs (PRL-PAs, 32%-51%) and growth hormone-secreting PAs (GH-PAs, 9%-11%) are the two most common subtypes accounting for a large percentage of functional PAs. Adrenocorticotropin-secreting PAs (ACTH-PAs, 3%-6%) and thyrotropin-secreting PAs (TSH-PAs, <1%) can cause obvious clinical symptoms, but their incidence is lower than that of PRL-PAs and GH-PAs [2, 5, 6]. Most gonadotropin adenomas (GON-PAs) and pluri-hormonal PAs are NFPAs. The majority of pituitary tumors are benign and exhibit arrested cell cycle as well as aberrant growth factor signaling [7, 8].

Transcription level alterations have been linked to abnormal hormone secretion in different subtypes of PAs [9]. In ACTH-PAs, the proopiomelanocortin (POMC), T-Box transcription factor 19 (TBX19, TPIT) and epidermal growth factor receptor (EGFR) genes are reportedly upregulated [10]; aberrant splicing of estrogen-related receptor gamma (ESRRG) leads to stronger binding to POU class 1 homeobox 1 (POU1F1, PIT-1) and excessive prolactin secretion [11]. Transcriptomic and epigenomic approaches have been employed to illustrate divergent patterns of gene expression in several subtypes of PAs [12]. However, the global changes in gene expression in PAs are still under investigation due to the lack of normal pituitary tissue as a control.

Genomic analyses have identified genes with somatic single-nucleotide variations (SNVs) in PAs, e.g., Ubiquitin-specific peptidase 8 (USP8) in ACTH-PAs, G protein α_s_ (GNAS) in GH-PAs and splicing factor 3b subunit 1 (SF3B1) in PRL-PAs [13–16]. However, these genomic studies suggested that PAs are associated with low mutation burdens; in addition, the frequencies of the three recurrent mutations were quite low, indicating a limited impact of SNVs on widespread gene expression alterations in PAs [12]. The impact of other genomic alterations on transcriptomic changes, such as copy number variations, remains to be investigated.

In this study, we performed whole-genome sequencing (WGS) and transcriptomic sequencing (RNA-seq) on PRL-PAs, GH-PAs and NFPAs to identify subtype-specific genomic and transcriptomic alterations. Normal pituitary tissues were also used to identify common gene expression abnormalities in PAs. Common gene expression alterations were detected in PAs, including genes in neuronal pathways and growth pathways. Widespread and unrecognized genomic copy number amplifications were identified in PRL-PAs, contributing to specific transcriptional activation in numerous genes and worse clinical outcomes of PRL-PA patients.

## RESULTS

### Transcriptional landscape of pituitary tumors

**Figure 1:**
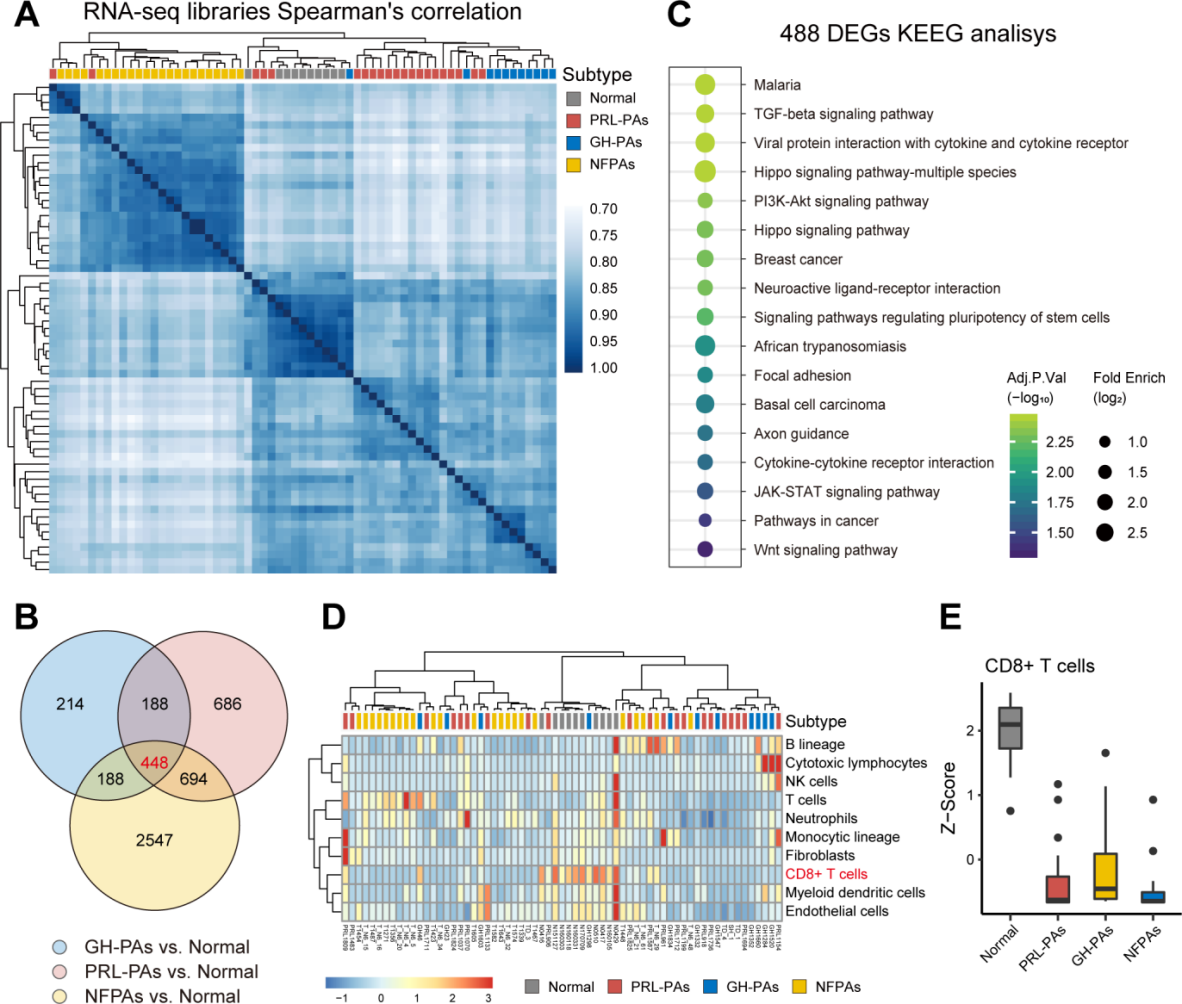
**The transcriptional landscape of PAs.** (**A**) Correlation heatmap of transcriptomic similarity among 21 PRL-PAs, 11 GH-PAs, 23 NFPAs and 9 normal pituitary tissues (Normal). Pituitary adenoma subtype is indicated by the color bar above the heatmap. (**B**) Venn diagram showing the intersection of DEGs among three subtypes of PAs vs. Normal. DEGs were identified by the R package DESeq2 under the cutoff of adjusted P value < 0.05. (**C**) KEGG pathway enrichment analysis of 448 overlapping DEGs, the dot plot shows pathways with an adjusted P value < 0.05. (**D**) The infiltration of eight subtypes of immune cell populations and two endothelial cell types in PA samples and normal pituitary tissues was evaluated using the expression levels of cell type specific markers using the MCP-counter [38]. The abundances of each cell types were normalized by z transformation across samples. (**E**) Boxplots of z score from Figure 1D showing the reduced infiltration of CD8^+^ T cells was reduced across PA subtypes compared to normal pituitary tissues.

We performed transcriptome sequencing (RNA-seq) on 54 PA samples (21 PRL-PAs, 11 GH-PAs, and 23 NFPAs) and 9 normal pituitary tissues (Supplementary Table 1). Hierarchical clustering showed that these specimens clustered according to their clinical groups, suggesting widespread transcriptomic alterations in PAs and across PA subtypes and that transcriptional signatures can be used for molecular classification of PA subtypes. The similarity heatmap suggests that NFPAs exhibit more dramatic transcriptomic alterations (Figure 1A). Consistently, the number of DEGs between NFPAs and other groups (3800~4200) was significantly larger than the number of DEGs between other comparison groups (Supplementary Figures 1, 2). This result suggests that despite a lack of abnormal hormone secretion, NFPAs are characterized by more widespread gene expression alterations.

A total of 448 common DEGs were found to be shared across PA subtypes compared to normal pituitary tissue (Figure 1B). These genes are enriched with neuronal pathways, including neural active ligand-receptor binding and axon guidance (Figure 1C). The common DEGs are also enriched in five growth factor signaling pathways (Wnt, TGF-β, PI3K-AKT, Hippo, and STAT3-JAK), all of which have been linked to PA pathogenesis or therapy [17–21]. These genes commonly changed across PA subtypes are also involved in cytokine production pathways (Figure 1C), consistent with decreased CD8^+^ T cell infiltration in both functional PAs and NFPAs (Figure 1D, 1E) and suggesting suppression of the immune response as a common etiology of PAs.

### Transcriptional signatures reveal altered pathways across PA subtypes

**Figure 2:**
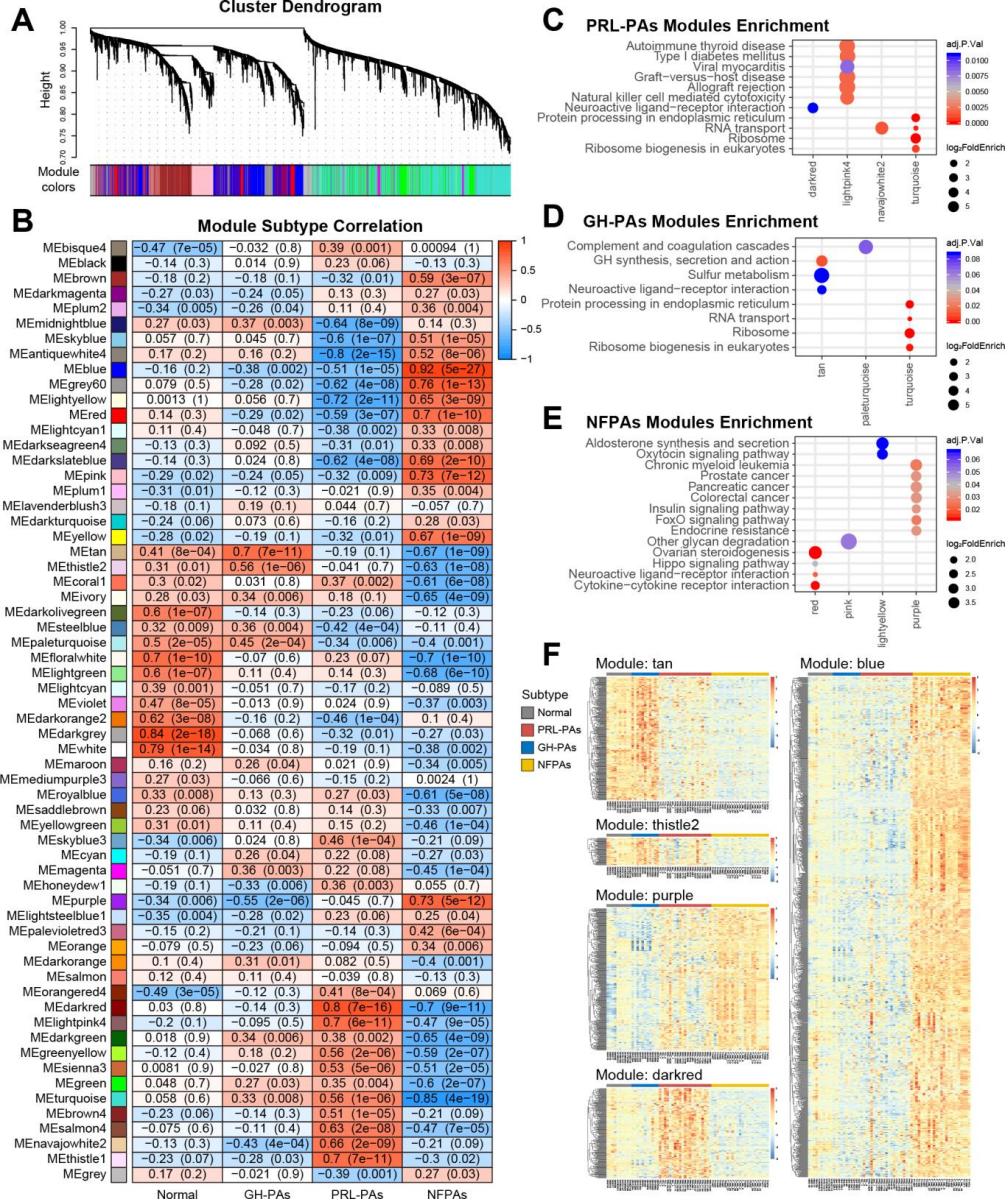
**Weighted gene correlation network analysis (WGCNA) of transcriptome data reveals subtype specific coexpression modules.** (**A**) WGCNA cluster dendrogram groups genes into distinct gene coexpression modules defined by dendrogram branch cutting. (**B**) Modules-subtype correlation heatmap of three PA subtypes and normal tissues. The cells in the heatmap were colored by the correlation between eigengene expression and each sample group, the correlation coefficients and P values were indicated in each cell. (**C**–**E**) KEGG pathway enrichment analysis of genes in the modules significantly associated with different PA subtypes. Adjusted P value < 0.05. (**F**) Expression profiles of genes in key modules associated with PA subtypes.

To identify transcriptional signatures associated with each PA subtype, 54 transcriptomic datasets were used to construct a weighted gene coexpression network [22] consisting of 62 modules with a size of 30~1500 genes (Figure 2A). Analysis of module trait association revealed modular alterations of gene expression in each subtype (Figure 2B and Supplementary Figure 3). The two abnormal hormone-secretion subtypes share some coexpression modules (e.g., MEturquoise, MEgreen and MEdarkgreen), and the module MEturquoise genes was enriched with pathways related to protein synthesis (Figure 2C, 2D), indicating that abnormal macromolecule biosynthesis may be the basis of aberrant hormone production and secretion. Several coexpression modules were significantly associated with the nonfunctional PA subtype, e.g., the Module purple showed enrichment of genes in the insulin signaling pathway (Figure 2E). As patients with hormone-abnormal PA usually develop insulin resistance and glucose abnormalities [23], activated insulin signaling in NFPAs might antagonize metabolic disorders, thus preventing excessive hormone secretion. The high expression of genes in subtype-correlated modules was confirmed by the heatmaps (Figure 2F).

### Genomic copy number amplifications causally involved in gene transcriptional activation in PRL-PAs

**Figure 3:**
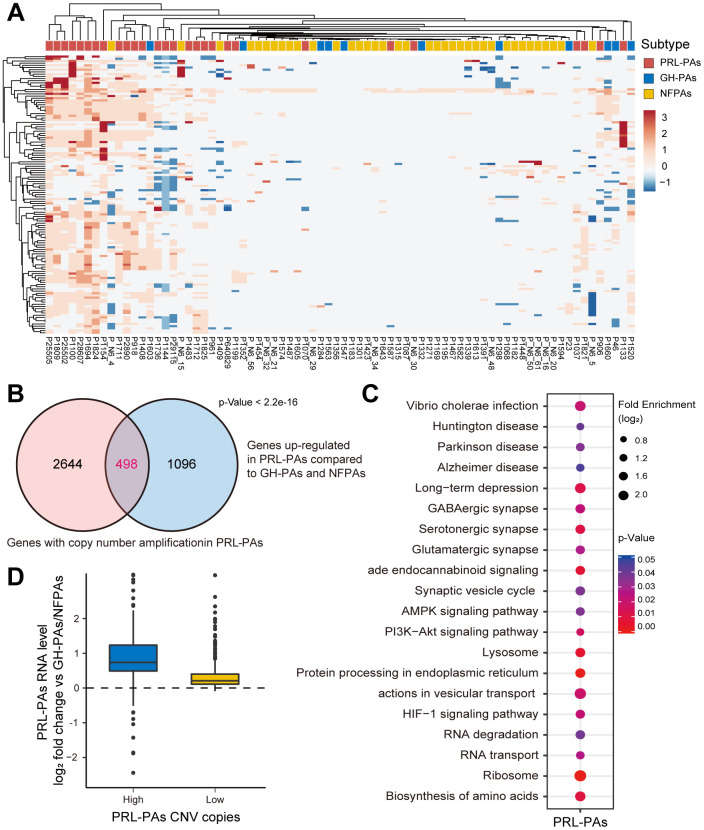
**Copy number amplifications in PRL-PAs cause gene transcriptional activation.** (**A**) The heatmap of CNV profiles across PA subtypes. The sample subtype was indicated by color bar above the heatmap, samples were grouped according to the similarity of CNV profiles. The heatmap was colored by CNVs, red indicates gain of copy number and blue indicates loss of copy number. Frequent copy number amplification was observed in PRL-PAs. (**B**) 498 genes with the copy number amplifications in PRL-PAs overlapped with up-regulated DEGs in the PRL-PAs compared to other subtypes. P value < 2.2e-16 by hypergeometric test. (**C**) KEGG pathway enrichment analysis of 498 up-regulated genes with both copy number amplifications and transcriptional up-regulation in PRL-PAs, the dot plot shows pathways with a P value < 0.05. (**D**) PRL-PAs samples were divided into two groups (high CNV and low CNV) according to the clustering results in (**A**). The boxplot shows the log2 expression fold-changes of PRL-PAs specific up-regulation genes relative to GH-PAs/NFPAs in the high CNV group and low CNV group. The high CNV group exhibited transcriptional up-regulation (Median log2 fold change ~0.75) while the low CNV group did not.

Next, we attempted to explore genomic alterations underlying the observed transcriptomic changes in PA patients. WGS data from 76 PA samples and paired blood samples were analyzed. We noticed a similar low mutation burden in these PA samples, consistent with previous observations. Some previously reported SNVs associated with PAs, such as GNAS and 1/11 in GH-PAs, were identified with a low frequency of occurrence (Supplementary Material 1). Strikingly, we found widespread and recurrent copy number variations, especially amplifications, in PRL-PAs. The high-CNV feature occurred in nearly half of the PRL-PA cases but rarely in other PA subtypes (Figure 3A). Specific genomic copy number amplification was not mentioned in a previous exome sequencing analysis of PAs, possibly because of the low number of PRL-PA patients involved.

The genes located in copy number amplification regions significantly overlapped with the genes specifically upregulated in PRL-PAs compared to other subtypes (Figure 3B), implying a role of genomic CNV in shaping transcriptomic alterations in PRL-PAs. The 498 overlapping genes were enriched in pathways downstream of mTOR signaling, including the biosynthesis of amino acids, lysosome pathway, ribosome and AMPK signaling (Figure 3C). Cyclin-dependent kinase 6 (CDK6) was also transcriptionally activated by copy number amplification, which suggests the role of genomic copy number variation in the out-of-control cell cycle and tumor progression of PAs.

We further divided PRL-PA patients into high-CNV and low-CNV groups according to the hierarchical clustering of CNV signatures. Consistent with the increase in copy number, the expression fold change of PRL-PA specific upregulated genes was significantly higher in the high-CNV group (Figure 3D), suggesting a causal role of CNVs in transcriptional changes in PRL-PA patients.

### Genomic copy number amplifications cause high prolactin production and activation of genes downstream of the mTOR signaling pathway

**Figure 4:**
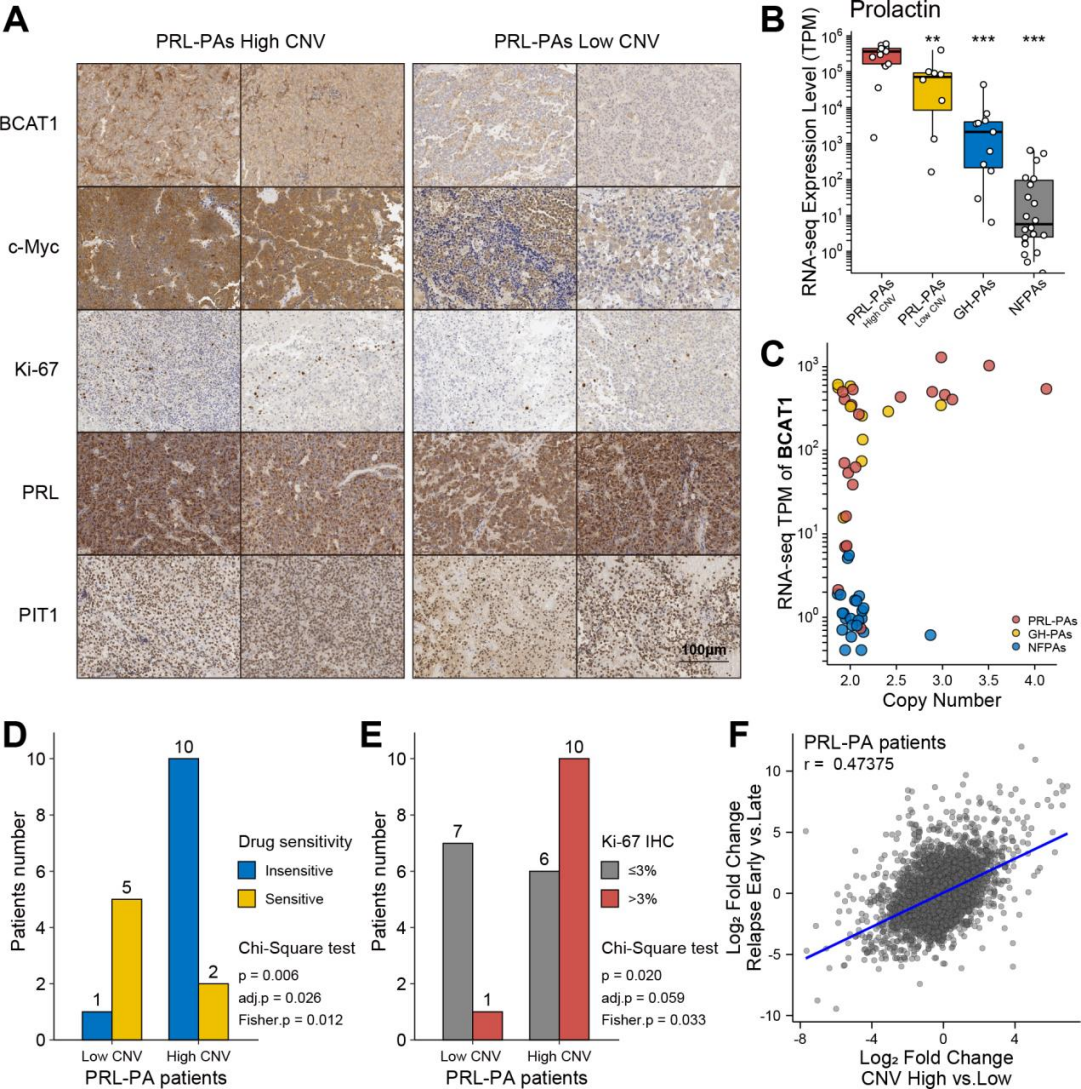
**Clinical relevance of genomic copy number variation in PRL-PAs.** (**A**) Immunohistochemistry of BCAT1, MYC, Ki-67, PRL, and PIT1 in high and low CNV PRL-PAs. ×100 magnification, the scale bar = 100 μm. (**B**) Prolactin expression levels (TPM) in PRL-PAs with high CNV, PRL-PAs with low CNV, GH-PAs and NFPAs. *P<0.05, **P<0.01, ***P<0.001 (**C**) Copy number and expression level (TPM) of BCAT1 in different PA subtypes. Both copy number and expression level of BCAT1 were increased in PRL-PAs. (**D**) PRL-PA patients in the high CNV group more frequently developed drug resistance. The yellow bar indicates the number of patients that are sensitive to BCT treatment, while the blue bar indicates number of patients that are insensitive to the same treatment. P value = 0.026, Chi-Square test. (**E**) High CNV group in PRL-PAs exhibits a higher degree of malignancy. The PA malignancy was defined by the number of positive Ki-67 foci in IHC. P value = 0.059, Chi-Square test. (**F**) The Scatter plot shows positive correlation (spearman correlation coefficient: 0.47) of transcriptomic alterations in high CNV group relative to low CNV group (x-axis) and relapsed PAs relative to un-relapsed PAs (y-axis).

To investigate the influence of genomic copy number amplification on the clinical outcomes of PRL-PA patients, we used an immunohistochemistry (IHC) staining approach to probe key genes under copy number amplifications in PRL-PAs. The copy number-amplified genes BCAT1 and MYC were more abundant in the high-CNV group of patients (Figure 4A). To further analyze the pathological impacts of genomic copy number variation, we surveyed expression of prolactin in patients in the high- and low-CNV groups and found prolactin expression to be 4-fold higher in the former group (Figure 4B). ErbB signaling is a determinant of prolactin production, and the downstream transcription factor of ErbB signaling, MYC, also exhibited a 4-fold increase in the high-CNV group. These results suggest a significant impact of genomic CNVs on the excessive production of prolactin in PRL-PA patients. The transcriptomic differences between the high- and low-CNV groups also involved numerous ribosome genes and branched-chain amino acid aminotransferase 1 (BCAT1), implicating activation of the mTOR signaling pathway in the high-CNV group (Figure 4C). BCAT1 undergoes correlated copy number amplifications and gene expression increments in PRL-PAs. Both BCAT1 and prolactin can activate the mTOR signaling pathway, which leads to excessive ribosome biogenesis.

### Genomic copy number amplifications are associated with worse prognosis

Elevated Ki-67 IHC staining in the PRL-PA high-CNV group suggests a worse clinical prognosis (Figure 4A and Table 1). Dopamine agonists (bromocriptine, BCT) are the first choice for PRL-PA treatment (not applicable to patients with rapid visual loss and visual field defects), followed by surgery, which can reduce tumor size and prolactin levels [4, 24]. However, drug resistance arises in a subset of patients, as indicated by a maintained prolactin level or tumor size. The frequency of BCT resistance in patients in the high-CNV group (BCT resistance: 10/12 v.s. BCT sensitive: 1/6; p = 0.006, chi-square test) was significantly higher than that in the low-CNV group (Figure 4D). The Ki-67 label index marks aggressive behavior in pituitary tumors, and the high-CNV group (Ki-67 positive ≥3%: 10/16 vs. Ki-67 positive < 3%: 1/8; P value = 0.02, chi-square test) was associated with a higher Ki-67 label index (Figure 4E). These data collectively support that copy number variations contribute to a worse prognosis in PRL-PA patients.

**Table 1:** Clinico-pathological characteristics of high- and low- CNV PRL-PAs.

**Sample**	**Volume^1^**	**Invasive/non-Invasive**	**CNV^2^**	**Drug resistance^3^**	**Ki-67 IHC^4^**
P906	Large	Invasive	High	Resistance	8%
P918	Large	non-Invasive	High	NA	<1%
P1133	Large	non-Invasive	High	NA	2%
P1154	Giant	Invasive	High	NA	>3%
P1037	Giant	Invasive	High	Resistance	3%
P1408	Giant	non-Invasive	High	Resistance	<1%
P1100	Large	non-Invasive	High	Resistance	<1%
P1694	Large	Invasive	High	NA	5-8%
P1711	Large	non-Invasive	High	NA	NA
P1712	Giant	Invasive	High	Resistance	6%
P1736	Giant	Invasive	High	Resistance	8%
P1809	Large	Invasive	High	Resistance	7%
P25502	Large	Invasive	High	Sensitive	3-5%
P28607	Large	Invasive	High	Resistance	1-2%
P2890	Large	non-Invasive	High	NA	2-3%
P1824	Giant	Invasive	High	Sensitive	5-10%
P25505	Large	Invasive	High	Resistance	<1%
P1821	Large	non-Invasive	High	Resistance	NA
P1070	Large	non-Invasive	Low	Sensitive	2%
P961	Giant	Invasive	Low	NA	<1%
P1483	Giant	Invasive	Low	Sensitive	<1%
P1587	Large	Invasive	Low	NA	NA
P_N6_30	Large	non-Invasive	Low	Sensitive	<1%
P1199	Large	Invasive	Low	Resistance	<1%
P1144	Large	Invasive	Low	Sensitive	<1%
P1825	Giant	Invasive	Low	NA	1%
P640829	Large	Invasive	Low	NA	NA
P29115	Large	Invasive	Low	Sensitive	2-5%

^1^ Tumor classification by volume, micro: <1 cm, large: 1-4 cm, giant: >4 cm.

^2^ According to the hierarchical clustering of CNV signatures.

^3^ Drug (BCT) resistance, NA: no drug therapy was administered preoperatively.

^4^ Ki-67 IHC staining, NA: not enough tissue.

The transcriptional signature related to relapse in PAs was determined by comparing patients who experienced early relapse (< 36 months) and those without any sign of relapse after a long period (> 60 months). Gene expression patterns in the high-CNV group in PRL-PA patients correlated with gene expression patterns that predict early relapse (Figure 4F), suggesting that the genes regulated by aberrant copy number in PRL-PAs are related to relapse.

## DISCUSSION

In this study, we profiled transcriptomic alterations in three major subtypes of Pas revealing shared and subtype-specific alterations. The clinical subtypes of PAs were well separated according to their transcriptional signatures in clustering analysis, suggesting that gene expression abnormalities are an essential part of PA etiology. Interestingly, we found that the PA subtypes with abnormal hormonal secretion were more similar to normal pituitary tissue samples and that the number of DEGs between samples of these functional subtypes and normal pituitary tissues samples was less than that of functional subtypes. These findings suggest that the nonfunctional subtype of PA undergoes more widespread gene expression alterations, as opposed to aberration of specific hormonal pathways.

We utilized WGCNA to generate coexpression networks in PAs and identified subtype-specific modules. The functional subtypes (GH-PAs and PRL-PAs) shared modules enriched in growth hormone pathways; PRL-PAs were also enriched in ribosome genes and the mitochondrial oxidative phosphorylation pathways, suggesting a high demand of energy metabolism in the PRL-PAs. The transcriptional alterations in nonfunctional PAs were enriched in autophagy genes, and induction of autophagy might be beneficial in limiting PA progression. Indeed, autophagic cell death mediates the effects of bromocriptine and temozolomide on PA [25–27]. Nevertheless, the specific roles of autophagy in antagonizing PA progression remain elusive.

Consistent with previous reports, we observed a lack of high-frequency recurrent mutations in PAs [12]. However, we do demonstrate frequent genomic copy number amplifications in PRL-PAs. Moreover, these genomic CNVs in PRL-PAs play a causal role in PA etiology, including prolactin production, proliferative ability, and drug resistance. The impact of copy number amplifications on PA etiology might be caused by its direct influence on abnormal gene expression upregulation, such as genes in ribosome biogenesis, growth signaling and HIF signaling pathways, which facilitate the hypoxic adaptative ability of PAs. The copy number amplification and upregulated expression of BCAT1 may play a central role in the activation of genes downstream of the mTOR pathway through a feedback loop involving prolactin [28, 29]. In addition, PRL-PA-specific upregulation of genes caused by copy number amplification includes chromogranin B (CHGB), which has been linked to tumor progression in PRL-PAs [30], further supporting the role of genomic copy number variation in PA progression.

Altogether, we investigated the genomic and transcriptomic correlation in PAs in the present study and demonstrated for the first time that high CNVs in PRL-PAs play an important role in tumor development and are significantly associated with poor prognosis. We believe the findings have clinical relevance in defining prognostic subgroups as well as implications for developing new targeted treatments for PRL-PA.

## MATERIALS AND METHODS

### Patients samples

All samples were obtained following transsphenoidal surgery performed at Beijing Tiantan Hospital from May 2013 to May 2017. The fresh tumor samples were stored in liquid nitrogen. 28 PRL-PAs, 11 GH-PAs and 37 NFPAs from the study population (age range, 20–75 years) were diagnosed according to clinical features and pathological findings. 9 normal pituitary glands were obtained from a donation program. The study protocols were approved by the Internal Review Board of Beijing Tiantan Hospital affiliated to Capital Medical University and conformed to the ethical guidelines of the Declaration of Helsinki (No. KY-2013-02).

### High-throughput sequencing

A total amount of 0.6 μg genomic DNA per sample was used as input material for DNA sample preparation. Sequencing libraries were generated using the Agilent SureSelect Human All Exon V6 kit (Agilent Technologies, CA, USA) following the manufacturer’s recommendations, and index codes were added to each sample. Clustering of the index-coded samples was performed using a cBot Cluster Generation System with a HiSeq PE Cluster Kit (Illumina, San Diego, CA, USA) according to the manufacturer’s instructions. After cluster generation, the DNA libraries were sequenced using the Illumina HiSeq platform, and 150-bp paired-end reads were generated. A total amount of 2 μg RNA per sample was used as input material for RNA sample preparations. Sequencing libraries were generated using NEBNext® UltraTM RNA Library Prep Kit for Illumina® (NEB, Ispawich, USA) following the manufacturer’s recommendations, and index codes were added to attribute sequences to each sample. Clustering of the index-coded samples was performed on a cBot Cluster Generation System using TruSeq PE Cluster Kit v3-cBot-HS (Illumina) according to the manufacturer’s instructions. After cluster generation, the library preparations were sequenced using the Illumina HiSeq platform, and 125-bp/150-bp paired-end reads were generated.

### Transcriptomic analysis and identification of differentially expressed genes

For RNA sequencing data, the paired-end clean reads were aligned to the human reference genome (hg19) using Hisat2 (v2.0.5) [31]. HTSeq (v 0.11.2) was used to count the read numbers mapped to each gene [32]. Hierarchical clustering analysis of all transcriptomic samples was performed using the pairwise similarity of each pair of samples determined by the Spearman correlation coefficient. Analysis of differentially expressed genes (DEGs) in each pair of comparisons was performed using the R package DESeq2 [33]. The P value of the differential test was corrected by a multiple hypothesis test, and DEGs were determined by controlling the FDR (false discovery rate) < 0.05.

### Genomic analysis

For WGS data, valid sequencing data were mapped to the reference human genome (UCSC hg19) by Burrows-Wheeler Aligner (BWA) software to obtain the original mapping results stored in BAM format [34]. If one or one paired read(s) were mapped to multiple positions, the strategy adopted by BWA is to choose the most likely placement. If two or more likely placements are presented, BWA selects one randomly. SAMtools and Picard (http://broadinstitute.github.io/picard/) were used to sort BAM files and perform duplicate marking, local realignment, and base quality recalibration to generate the final BAM file. GATK (v3.4) software 28 was employed for SNP calling. Genome regions with significant amplification or deletion in the samples were evaluated by GISTIC [35], and regions with high frequencies were screened, namely, recurrent CNV regions. The higher the GISTIC score is, the higher is the CNV frequency in this area.

### Pathway enrichment analysis

KEGG pathway enrichment for functional analysis of gene lists was performed using the clusterprofiler package under R software (version 3.6.0) [36]. The significance of the enrichment analysis was defined using a hypergeometric test, and the resulting P values were corrected for multiple hypothesis testing with the BH (Benjamini & Hochberg, 1995) method [37]. The final reported enriched terms and pathways were filtered according to adjusted P values < 0.05 or P value < 0.05.

### Weighted gene expression network analysis (WGCNA)

The PA coexpression network was constructed using the R package WGCNA (v 1.69) [22]. Biweight midcorrelations between each gene pair were calculated to build an adjacency matrix using the formula: adjacency = (0.5 * (1+cor)) power. A thresholding power of 14 was chosen, and the resulting adjacency matrix was converted to a topological overlap (TO) matrix via the TOM similarity algorithm. The genes were hierarchically clustered based on TO similarity. Modules were assigned by the dynamic tree-cutting algorithm with default parameters. Modules were correlated with each group of samples to identify subtype-specific modules, and the correlation coefficients and P values are indicated in figures.

### Immunohistochemistry staining

Tissue from PRL-PAs was fixed in 10% formalin and embedded in paraffin. Three core biopsies (2.0 mm in diameter) were selected from the paraffin-embedded tissue and transferred to tissue microarrays using a semiautomated system (Aphelys MiniCore, Mitogen, UK). The microarrays were cut into 4-μm sections and incubated with anti-BCAT1 (rabbit monoclonal, 1:600, ab197941, Abcam), anti-c-Myc (rabbit monoclonal, 1:1000, ab32072, Abcam), anti-Ki-67 (rabbit monoclonal, 1:100, ab16667, Abcam), anti-PRL (rabbit polyclonal, 1:300, ab188229, Abcam), and anti-PIT1 (mouse monoclonal, 1:500, sc393943, Santa-Cruz) primary antibodies. Staining intensity was scored as follows: 0, no staining: 1, weak; 2, moderate; and 3, strong staining. An H-score was calculated based on the percentage of positively stained cells at each intensity level using the following formula: [1 × (% weakly stained cells) + 2 × (% moderately stained) + 3 × (% strongly stained cells)].

### Statistical analysis

Chi test and Fisher's exact test were employed to determine the significance of categorical variables. Comparisons between two groups were performed using Student’s unpaired two-tailed t-test. A P value ≤0.05 and/or adjusted P value ≤0.05 was considered statistically significant.

### Ethics approval and consent to participate

The study protocols were approved by the Internal Review Board of Beijing Tiantan Hospital, which was affiliated to Capital Medical University, and conformed to the ethical guidelines of the Declaration of Helsinki (No. KY2016-035-01).

## Supplementary Material

Supplementary Figures

Supplementary Table 1

Supplementary Material 1
