# Supplementary material for: Genomic and transcriptomic analysis of pituitary adenomas reveals the impacts of copy number variations on gene expression and clinical prognosis among prolactin-secreting subtype
Source: Aging (Albany NY). 2020 Dec 19;13(1):1276–93. doi: 10.18632/aging.202304 (PMC7834992; doi:10.18632/aging.202304)
Supplement: Supplementary Figures [file aging-13-202304-s001.pdf]

SUPPLEMENTARY FIGURES

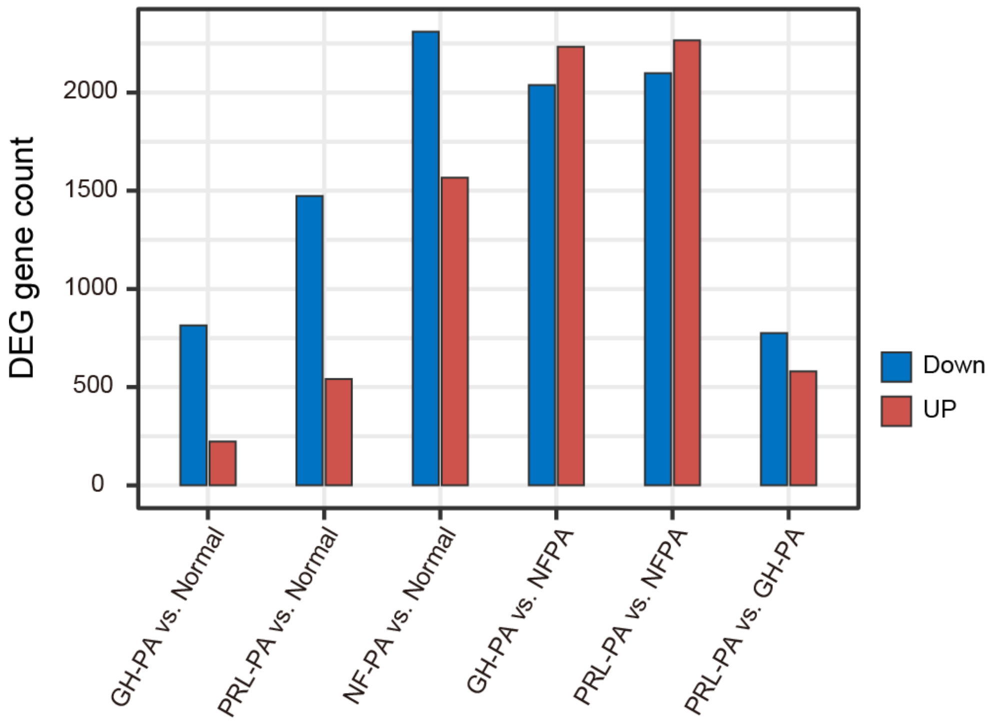

Supplementary Figure 1. Comparison of the number of upregulated DEGs (Red) and downregulated DEGs (Blue) in 6 pairwise DEGs analysis. DESeq2, FDR < 0.05.

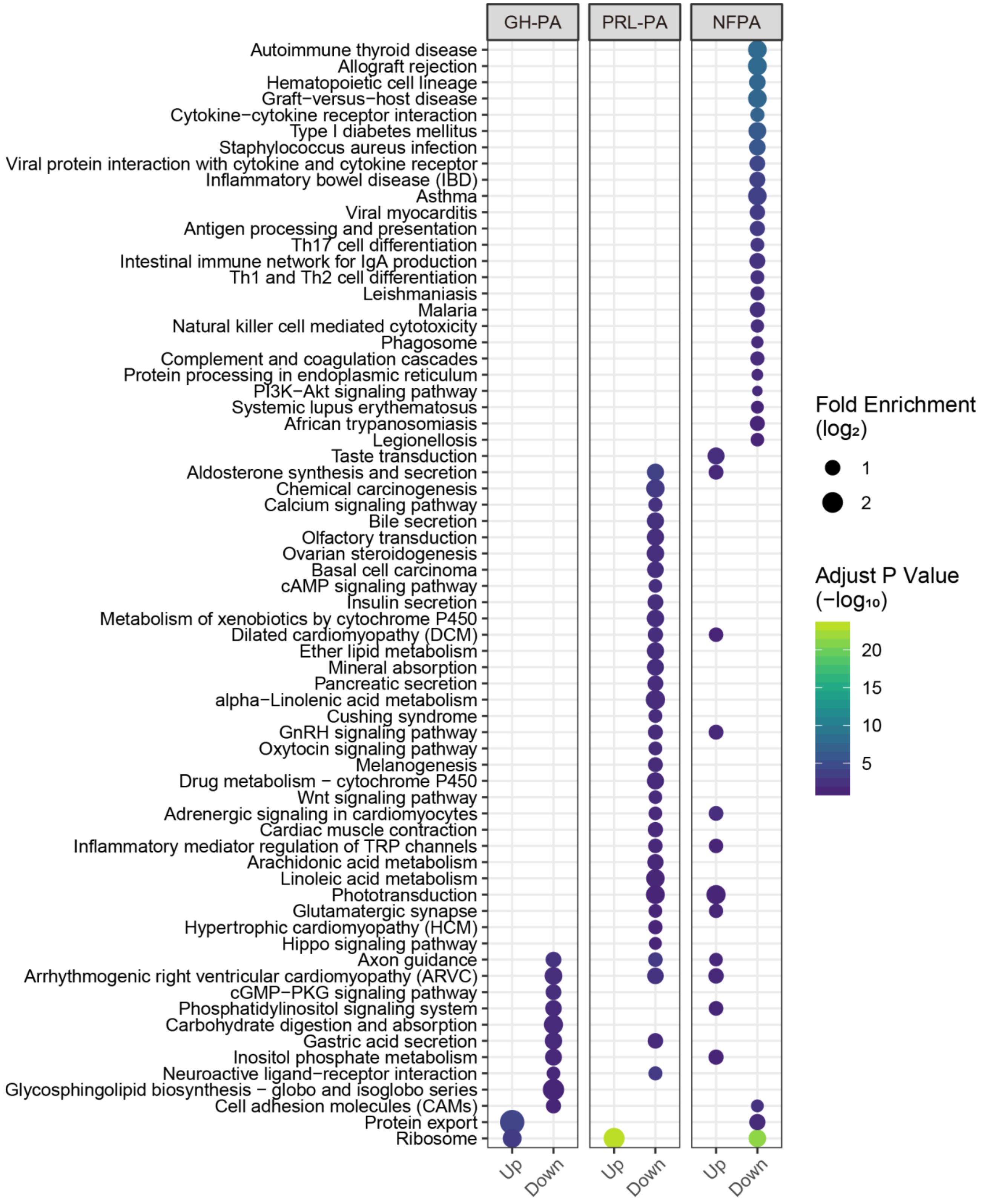

Supplementary Figure 2. KEGG pathway enrichment analysis of genes among three PA subtypes vs. Normal. Adjusted P value < 0.05.

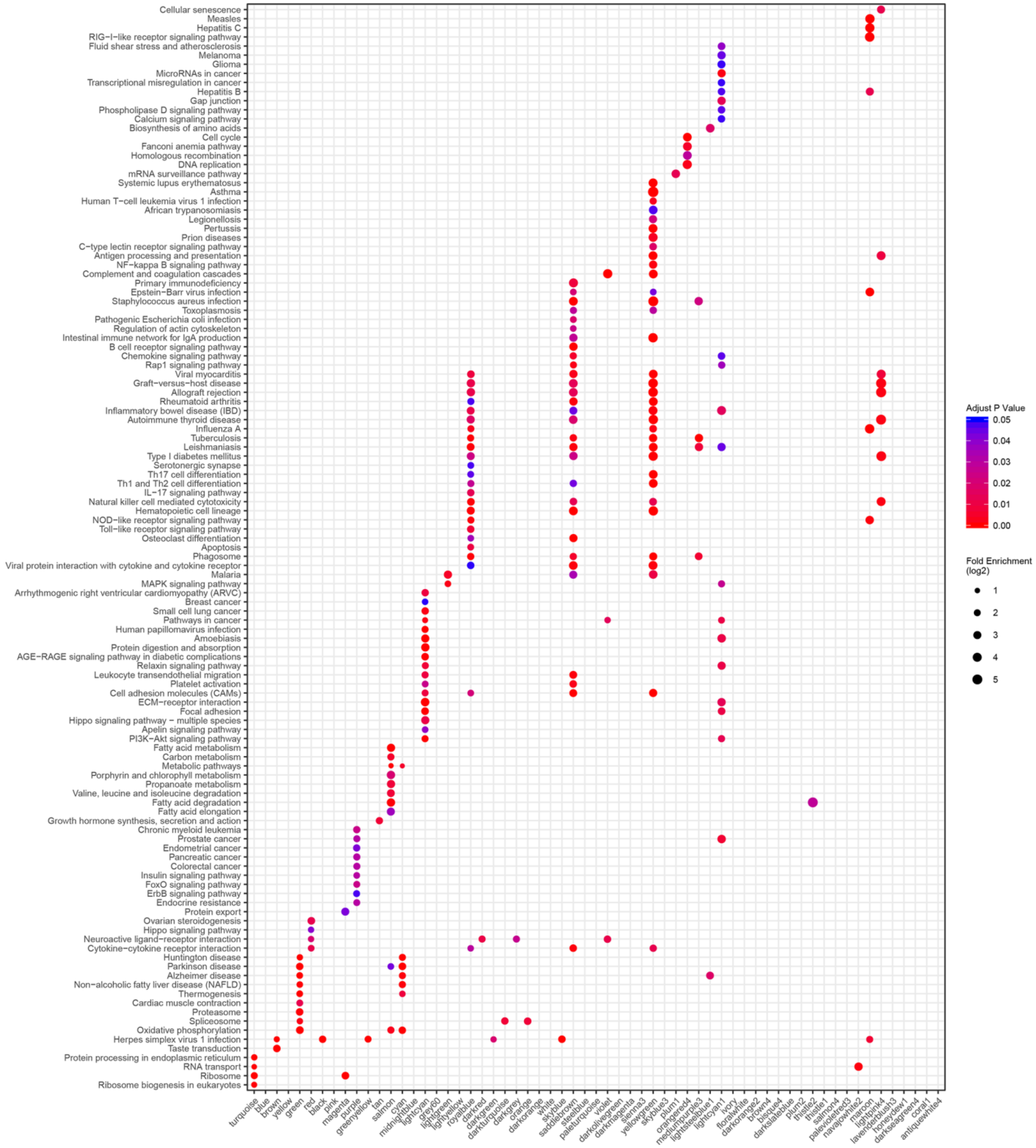

Supplementary Figure 3. KEGG pathway enrichment analysis of genes in the modules significantly associated with different PA subtypes. Adjusted P value < 0.05.
