# Supplementary material for: Genomic and transcriptomic analysis of pituitary adenomas reveals the impacts of copy number variations on gene expression and clinical prognosis among prolactin-secreting subtype
Source: Aging (Albany NY). 2020 Dec 19;13(1):1276–93. doi: 10.18632/aging.202304 (PMC7834992; doi:10.18632/aging.202304)
Supplement: Supplementary Table 1 [file aging-13-202304-s002.pdf]

SUPPLEMENTARY TABLE

Supplementary Table 1. Clinical features of 76 PAs.

Sample	Gender	Age (Y)	Clinical Type	Volume[1]	Invasive/non-Invasive	PFS Time[2]	Relapse[2]	Sequencing strategy
P906	F	25	PRL-PAs	Large	Invasive	81	N	WGS+RNA-seq
P918	M	49	PRL-PAs	Large	non-Invasive	80	N	WGS+RNA-seq
P1133	F	61	PRL-PAs	Large	non-Invasive	66	N	WGS+RNA-seq
P1154	F	46	PRL-PAs	Giant	Invasive	65	N	WGS+RNA-seq
P1037	M	43	PRL-PAs	Giant	Invasive	37	Y	WGS+RNA-seq
P1408	M	45	PRL-PAs	Giant	non-Invasive	52	N	WGS
P1100	M	37	PRL-PAs	Large	non-Invasive	41	Y	WGS
P1694	F	44	PRL-PAs	Large	Invasive	39	N	WGS+RNA-seq
P1711	F	30	PRL-PAs	Large	non-Invasive	-	-	WGS+RNA-seq
P1712	M	31	PRL-PAs	Giant	Invasive	8	Y	WGS+RNA-seq
P1736	M	55	PRL-PAs	Giant	Invasive	17	Y	WGS+RNA-seq
P1809	F	20	PRL-PAs	Large	Invasive	34	N	WGS+RNA-seq
P25502	M	65	PRL-PAs	Large	Invasive	-	-	WGS+RNA-seq
P28607	F	28	PRL-PAs	Large	Invasive	-	-	WGS+RNA-seq
P2890	F	15	PRL-PAs	Large	non-Invasive	-	-	WGS+RNA-seq
P1824	M	22	PRL-PAs	Giant	Invasive	33	N	WGS+RNA-seq
P25505	M	27	PRL-PAs	Large	Invasive	-	-	WGS
P1821	F	22	PRL-PAs	Large	non-Invasive	-	-	WGS
P1070	F	50	PRL-PAs	Large	non-Invasive	71	N	WGS+RNA-seq
P961	M	42	PRL-PAs	Giant	Invasive	53	Y	WGS+RNA-seq
P1483	M	20	PRL-PAs	Giant	Invasive	49	N	WGS+RNA-seq
P1587	F	26	PRL-PAs	Large	Invasive	44	N	WGS+RNA-seq
P_N6_30	F	48	PRL-PAs	Large	non-Invasive	52	N	WGS
P1199	F	41	PRL-PAs	Large	Invasive	4	Y	WGS+RNA-seq
P1144	M	48	PRL-PAs	Large	Invasive	66	N	WGS
P1825	F	38	PRL-PAs	Giant	Invasive	33	N	WGS+RNA-seq
P640829	M	43	PRL-PAs	Large	Invasive	-	-	WGS+RNA-seq
P29115	M	37	PRL-PAs	Large	Invasive	-	-	WGS
P1087	M	46	NFPAs	Giant	Invasive	25	Y	WGS
P1169	F	57	NFPAs	Giant	Invasive	65	N	WGS
P1271	M	56	NFPAs	Large	non-Invasive	59	N	WGS+RNA-seq
P1301	F	55	NFPAs	Large	non-Invasive	14	Y	WGS
P1315	F	50	NFPAs	Large	non-Invasive	57	N	WGS
P1339	M	43	NFPAs	Large	Invasive	55	N	WGS+RNA-seq
P1356	M	47	NFPAs	Large	Invasive	36	Y	WGS+RNA-seq
P1391	M	51	NFPAs	Large	non-Invasive	52	N	WGS
P1409	M	60	NFPAs	Large	Invasive	52	N	WGS
P1423	F	66	NFPAs	Large	non-Invasive	51	N	WGS+RNA-seq
P1448	F	49	NFPAs	Giant	Invasive	44	Y	WGS+RNA-seq
P1454	F	71	NFPAs	Giant	non-Invasive	50	N	WGS+RNA-seq
P1467	F	54	NFPAs	Giant	Invasive	24	Y	WGS+RNA-seq
P1487	M	65	NFPAs	Giant	Invasive	49	N	WGS+RNA-seq
P1574	M	65	NFPAs	Giant	Invasive	3	Y	WGS+RNA-seq
P1582	M	66	NFPAs	Giant	Invasive	45	N	WGS+RNA-seq
P1594	M	67	NFPAs	Large	non-Invasive	44	N	WGS
P1605	F	44	NFPAs	Large	non-Invasive	44	N	WGS+RNA-seq
P1613	M	51	NFPAs	Large	Invasive	43	N	WGS
P1643	M	43	NFPAs	Large	non-Invasive	36	Y	WGS+RNA-seq
P_N6_15	M	49	NFPAs	Giant	Invasive	55	N	WGS+RNA-seq
P_N6_16	M	34	NFPAs	Giant	Invasive	55	N	WGS+RNA-seq
P_N6_20	M	61	NFPAs	Giant	Invasive	54	N	WGS+RNA-seq
P_N6_21	F	51	NFPAs	Large	Invasive	54	N	WGS+RNA-seq
P_N6_29	M	47	NFPAs	Large	non-Invasive	52	N	WGS+RNA-seq

P_N6_32	M	64	NFPAs	Giant	Invasive	52	N	WGS+RNA-seq
P_N6_34	M	55	NFPAs	Giant	Invasive	52	N	WGS+RNA-seq
P_N6_4	F	32	NFPAs	Large	Invasive	57	N	WGS+RNA-seq
P_N6_48	M	64	NFPAs	Giant	Invasive	51	N	WGS+RNA-seq
P_N6_5	F	35	NFPAs	Giant	Invasive	57	N	WGS+RNA-seq
P_N6_50	F	41	NFPAs	Large	Invasive	51	N	WGS
P_N6_56	M	45	NFPAs	Large	Invasive	51	N	WGS
P_N6_61	F	49	NFPAs	Giant	Invasive	49	N	WGS+RNA-seq
P1183	F	57	NFPAs	Giant	Invasive	66	N	WGS
P1068	M	48	NFPAs	Giant	Invasive	19	Y	WGS
P1182	M	67	NFPAs	Large	Invasive	66	N	WGS
P1195	M	35	NFPAs	Large	non-Invasive	65	N	WGS
P1284	F	21	GH-PAs	Giant	Invasive	12	Y	WGS+RNA-seq
P1298	F	69	GH-PAs	Large	non-Invasive	57	N	WGS+RNA-seq
P1332	M	57	GH-PAs	Giant	Invasive	14	N	WGS+RNA-seq
P1352	M	31	GH-PAs	Giant	Invasive	3	Y	WGS+RNA-seq
P23	F	51	GH-PAs	Giant	Invasive	4	Y	WGS+RNA-seq
P46	F	44	GH-PAs	Large	non-Invasive	51	N	WGS+RNA-seq
P1603	M	45	GH-PAs	Large	non-Invasive	44	N	WGS+RNA-seq
P1563	M	32	GH-PAs	Large	Invasive	12	Y	WGS
P1547	F	33	GH-PAs	Large	non-Invasive	46	N	WGS+RNA-seq
P1520	M	21	GH-PAs	Giant	Invasive	17	Y	WGS+RNA-seq
P1660	F	51	GH-PAs	Large	non-Invasive	41	N	WGS+RNA-seq

[1] Tumor classification by volume, micro: <1 cm, large: 1-4 cm, giant: >4 cm.
[2] Patients were lost to follow-up: -
